# Improving resolution in multidimensional NMR using random quadrature detection with compressed sensing reconstruction

**DOI:** 10.1007/s10858-016-0062-9

**Published:** 2016-09-20

**Authors:** M. J. Bostock, D. J. Holland, D. Nietlispach

**Affiliations:** 1 Department of Biochemistry, University of Cambridge, 80 Tennis Court Road, Old Addenbrooke’s Site, Cambridge, CB2 1GA UK; 2 Chemical and Process Engineering Department, University of Canterbury, Christchurch, New Zealand

**Keywords:** Compressed sensing, Non-uniform sampling, $l_{1}$-norm minimisation, NMR spectroscopy, Random quadrature detection (RQD), Gradient selection, CS_RQD_

## Abstract

NMR spectroscopy is central to atomic resolution studies in biology and chemistry. Key to this approach are multidimensional experiments. Obtaining such experiments with sufficient resolution, however, is a slow process, in part since each time increment in every indirect dimension needs to be recorded twice, in quadrature. We introduce a modified compressed sensing (CS) algorithm enabling reconstruction of data acquired with random acquisition of quadrature components in gradient-selection NMR. We name this approach random quadrature detection (RQD). Gradient-selection experiments are essential to the success of modern NMR and with RQD, a 50 % reduction in the number of data points per indirect dimension is possible, by only acquiring one quadrature component per time point. Using our algorithm (CS_RQD_), high quality reconstructions are achieved. RQD is modular and combined with non-uniform sampling we show that this provides increased flexibility in designing sampling schedules leading to improved resolution with increasing benefits as dimensionality of experiments increases, with particular advantages for 4- and higher dimensional experiments.

**Electronic supplementary material:**

The online version of this article (doi:10.1007/s10858-016-0062-9) contains supplementary material, which is available to authorized users.

## Introduction

Multidimensional ($n{\text{D}}$) NMR experiments are indispensable for high resolution NMR spectroscopy studies of macromolecules in biology and chemistry. However, obtaining adequate resolution requires lengthy data sampling that may compromise the achievable sensitivity and lead to extended data collection times.

An area of intense interest for fast NMR spectroscopy involves non-uniform sampling (NUS) of the time domains enabling reduction of the number of acquired time points in all indirect dimensions (Barna et al. 1987; Mobli and Hoch 2014). NUS may be used to improve sensitivity and resolution of NMR experiments compared to their fully sampled equivalents, however the Fast Fourier Transform (FFT) cannot be used to reconstruct the frequency domain spectrum (Palmer et al. 2015). A multitude of different reconstruction methods is available (Orekhov et al. 2001; Kupče and Freeman 2003; Atreya and Szyperski 2004; Tugarinov et al. 2005; Marion 2005; Kazimierczuk et al. 2006; Coggins and Zhou 2008; Matsuki et al. 2009), and recently compressed sensing based techniques (CS) have become popular (Kazimierczuk and Orekhov 2011; Holland et al. 2011; Hyberts et al. 2012).

Nevertheless, despite the improvements introduced by NUS approaches, the $n - 1$ indirect time dimensions of an $n{\text{D}}$ NMR experiment still need to be recorded in quadrature in order to generate high resolution spectra with signals sign-discriminated in frequency and absorptive in lineshape (Keeler and Neuhaus 1985; Ernst et al. 1990). Quadrature detection is very costly, requiring recording of two data points per indirect time increment, increasing the data collection time by a factor of $2^{n - 1}$ and further compromising the achievable spectral resolution. Maciejewski et al. (2011) suggested random acquisition of phase components (random phase detection (RPD)) with Maximum Entropy (MaxEnt) reconstruction as a sampling reduction strategy for amplitude modulated data, using a partial-component sampling scheme (Schuyler et al. 2015). Although in theory partial-component sampling (recording less than $2^{n - 1}$ quadrature components) is applicable to any NMR experiment, in practice, due to the lack of a suitable reconstruction method, this approach is not available to the majority of modern $n{\text{D}}$ NMR spectroscopy experiments, which typically use gradient-enhanced P- and N-type coherence order selection (so called gradient-selection or phase modulation) (see Theory section). Gradient-selection experiments are prevalent in NMR due to their superior artifact suppression and efficient reduction of large unwanted signals. Amongst the crucial experiments inaccessible to the RPD methodology is the [^1^H,^15^N]-TROSY class (Pervushin et al. 1997; Salzmann et al. 1998), which is instrumental for the study of large biomacromolecules.

We introduce a new CS-based algorithm (CS_RQD_) using a modified version of our in-house developed CS reconstruction method (Bostock et al. 2012), which enables reconstruction of data recorded with a partial-component sampling schedule using either amplitude or phase modulation and name this data reduction strategy random quadrature detection (RQD). Reconstruction of RQD data with CS_RQD_ is applicable to the full range of multidimensional NMR experiments, including those with gradient-enhanced coherence order selection and removes the need for complete quadrature detection in such experiments. The number of data points required is then reduced by a factor of two for every indirect time domain, which is achieved by acquiring only one quadrature component per time increment, with the detected component selected at random. Biomolecular NMR experiments are often limited by sensitivity and therefore require longer recording times; compared to full sampling, RQD enables sampling of the indirect dimensions with superior spectral resolution without the need to increase recording times.

Many NMR experiments are typically already recorded with NUS in order to improve resolution and/or sensitivity. The RQD approach is modular and can be combined with traditional NUS sampling. We show that the combination of RQD and NUS allows increased time-point sampling for a given sampling fraction compared to traditional full-component NUS, which may provide increased resolution and improved reconstruction properties; the benefits of RQD scale with dimensionality.

Consequently, RQD represents a key recording strategy suitable for all types of multidimensional NMR experiments with the potential to accelerate the sampling or improve resolution and reconstruction properties of every available indirect time domain.

## Theory

### Compressed sensing reconstruction of NUS data

Compressed sensing (CS) reconstructions have recently become popular in NMR spectroscopy for accurate and rapid reconstruction of NUS datasets using either convex $\ell_{1}$-norm minimization e.g. iterative thresholding (IT) (Kazimierczuk and Orekhov 2011; Holland et al. 2011; Hyberts et al. 2012) or non-convex approaches using $\ell_{p \to 0}$ minimisation (Kazimierczuk and Orekhov 2011).

Compressed sensing theory (Logan 1965; Candès et al. 2006; Donoho 2006) describes an approach for solving the system of linear equations

1 $${\mathbf{Ax}} = {\mathbf{b}}$$

where ${\mathbf{A}}$ is an $M \times N$ matrix and ${\mathbf{x}}$ is a vector of length $N$ to be recovered from a set of measurements, ${\mathbf{b}}$, with $M < N$. For NMR spectroscopy, ${\mathbf{A}}$ is the inverse Fourier transform at the points sampled, ${\mathbf{b}}$ is the time-domain data and ${\mathbf{x}}$ is the frequency domain spectrum. In this case, the equations are underdetermined and (1) has infinitely many solutions. The optimal solution can be obtained by finding the sparsest solution which is consistent with the measured data, i.e. minimising the $\ell_{0}$-norm, a pseudo-norm defined by:

2 $$||\mathbf{x}||_{0} = \mathop \sum \limits_{i} \,\left| {x_{i}} \right|^{0}$$

where $0^{0} = 0$ (Donoho 2006). However, this is computationally intractable (Natarajan 1995). Compressed sensing theory shows that minimising the $\ell_{1}$-norm, which can be carried out using standard linear processing, can achieve the same result provided the solution is sufficiently sparse:

3 $$\mathop {\hbox{min} }\limits_{{\mathbf{x}}} {||\mathbf{x}}||_{1} \varvec{ }{\text{subject to}}\, {\mathbf{Ax}} = {\mathbf{b}}$$

where

4 $${||\mathbf{x}||}_{1} = \mathop \sum \limits_{i} \,\left| {x_{i} } \right|$$

Non-convex minimisations solve an $\ell_{p}$-norm with $p > 0$ where $p$ is reduced with successive iterations:

5 $${||\mathbf{x}||}_{p} = \left( {\mathop \sum \limits_{i} \left| {x_{i} } \right|^{p} } \right)^{1{\text{/}}{p}}$$

For data containing noise, or which is compressible but not sparse, the constraint in (3) is relaxed for example to:

6 $$\mathop {\hbox{min} }\limits_{{\mathbf{x}}} {||\mathbf{x}||}_{1} \varvec{ }{\text{subject to}}\,\varvec{ }{||\mathbf{Ax}} - {\mathbf{b}||}_{2} \le\delta$$

where $\delta$ is an estimate of the noise level.

Compressed sensing requires data to be sparse in some basis e.g. the frequency domain for NMR spectra, and to have incoherent sampling with respect to that basis, achieved by selection of an appropriate sampling schedule.

NMR experiments can be successfully undersampled in the indirect time-domains using non-uniform sampling (Barna et al. 1987; Schmieder et al. 1994; Rovnyak et al. 2004), which may be represented as follows for a one-dimensional vector (Maciejewski et al. 2011):

7 $$\begin{aligned} &z_{j} = x_{j} + iy_{j} , \quad {\text{for}} \,\, j = 0, \ldots N - 1 \hfill \\ &z_{j}^{\text{NUS}} = \left\{ {\begin{array}{*{20}c} 0 \\ {x_{j} + iy_{j} } \\ \end{array} } \right.\begin{array}{*{20}c} { {\text{if}}} \\ { {\text{if}}} \\ \end{array} \begin{array}{*{20}c} { p_{j} = 0} \\ { p_{j} = 1} \\ \end{array} \hfill \\ \end{aligned}$$

where $j$ represents the time increments and ${\mathbf{p}}$ is the sampling vector i.e. $p_{j} = 1$ represents sampled points. Strictly speaking, for $p_{j} = 0,$ the point is skipped, i.e. no data is acquired. Thus ${\mathbf{z}}$ has length given by $\mathop \sum \limits_{j} p_{j}$.

### Compressed sensing reconstruction of RQD data

As well as the requirement to sample to long time points to achieve high resolution in multiple indirect dimensions, NMR spectra also require frequency discrimination and signals with a pure, absorptive, phase. This is achieved using quadrature detection, acquiring two data points per time increment. The total number of points acquired is

8 $$2^{{n - 1}} \times k_{1} \times k_{2} \times k_{3} \times \ldots \times k_{n}$$

where $k_{n}$ is the number of points in the $n^{\text{th}}$ dimension, and $2^{n - 1}$ results from quadrature detection of the $n - 1$ indirect dimensions. Quadrature detection may be achieved using amplitude modulated data, phase modulated data or by oversampling by a factor of two in each indirect dimension (the time-proportional phase incrementation method (TPPI) (Marion and Wüthrich 1983)). In each case, quadrature detection requires a factor of two increase in the number of points per indirect dimension.

Random acquisition of quadrature components, which we generalise as random quadrature detection (RQD) for NMR may be represented in a similar manner to full-component NUS data (7) according to hypercomplex notation (Delsuc 1988; Maciejewski et al. 2011). For a two dimensional experiment, a matrix of hypercomplex points, ${\mathbf{z}}$, is acquired:

9 $$z_{{j_{1} ,\,j_{2} }} = x_{{j_{1} ,\,j_{2} }} + i_{1} y_{{j_{1} ,\,j_{2} }} + i_{2} r_{{j_{1},\,j_{2} }} + i_{1} i_{2} s_{{j_{1} ,\,j_{2} }}$$

where

$$i_{1}^{2} = i_{2}^{2} = - 1$$

$$i_{1} \cdot i_{2} = i_{2} \cdot i_{1}$$

Assuming the directly acquired dimension is fully sampled as is typically the case, RQD sampling is only implemented in the indirect dimensions; for a 2D this is represented as follows:

10 $$z_{j}^{{{\text{RQD}}}} = \left\{\begin{array}{ll} x_{{{j}_{1} ,\,j_{2} }}+ i_{1} y_{{j_{1} ,\,j_{2} }}&{\text{if }} p_{{j_{1} ,\,j_{2} }} = 0 \\i_{2} r_{{j_{1},\,j_{2} }} +i_{1} i_{2} s_{{j_{1},\,j_{2} }}&{\text{if }} p_{{j_{1},\,j_{2} }} = 1\end{array} \right.$$

Similar to (7) for $p_{{j_{1},\, j_{2} }} = 0,$ the point is skipped, i.e. no data is acquired.

#### Amplitude modulated quadrature detection

Using the States (States et al. 1982) or States-TPPI (Marion et al. 1989) protocol the two quadrature components are represented by cosine and sine modulated datasets. In this case, both components generate an absorption mode spectrum, but without sign discrimination. The random phase detection (RPD) approach, demonstrated using MaxEnt reconstruction (Maciejewski et al. 2011) acquires one phase component for each time-point, selecting either the cosine or sine component at random. This approach is equally possible with standard CS reconstruction solving (6) where ${\mathbf{b}}$ represents cosine/sine type data (see Results).

#### Phase modulated quadrature detection (gradient-enhanced spectroscopy)

In contrast, phase modulated data obtained from gradient coherence order selection experiments shows frequency encoding either as ${ \exp }\left( { - i\Omega t_{n} } \right)$, N-type (echo) or ${ \exp }\left( { + i\Omega t_{n} } \right)$, P-type (anti-echo) coherence, where $\Omega$ is the offset frequency and $t_{n}$ the time-evolution in the $n^{\text{th}}$ indirect dimension. Such datasets give rise to frequency discriminated spectra with each sub-type generating peaks with a phase-twist lineshape i.e. a mixture of absorptive and dispersive components, unsuitable for high-resolution NMR work. Acquiring both components and converting them via linear combination to amplitude-modulated data ($S_{ \cos }$ and $S_{ \sin }$) generates pure absorption spectra (Davis et al. 1992):

11 $$\begin{aligned} &S_{ \cos } \left( t \right) = \left( {{ \exp }\left( {i\Omega t} \right) + { \exp }\left( { - i\Omega t} \right)} \right)/2 = \left( {S_\text{P} + S_\text{N} } \right)/2 \hfill \\ & S_{ \sin } \left( t \right) = \left( {{ \exp }\left( {i\Omega t} \right) - { \exp }\left( { - i\Omega t} \right)} \right)/2i = \left( {S_\text{P} - S_\text{N} } \right)/2i \hfill \\ \end{aligned}$$

Random acquisition of either the P-type or N-type component for each time increment is also possible, but this cannot be processed using the standard CS approach due to the intense artifacts generated by the phase-twist lineshape. However, (11) represents a linear transformation of the P-/N-type data. Therefore, this transformation can be built into the compressed sensing reconstruction by introducing an additional matrix, ${\mathbf{U}}$, which converts data of the form $S_{ \cos } ,S_{ \sin }$ to $S_\text{P} ,S_\text{N}$ at each iteration, ensuring that the reconstructed frequency domain data at each iteration, ${\mathbf{x}}$, is constrained via the $\ell_{2}$-norm term to the raw P-/N-type data, ${\mathbf{b}}_\text{PN}$. Equation (6) is reformulated to include this function:

12 $$\mathop {\hbox{min} }\limits_{{\mathbf{x}}} {||\mathbf{x}||}_{1} \varvec{ }{\text{subject to}}\,\varvec{ }{||\mathbf{UAx}} - {\mathbf{b}}_{\text{PN}}||_{2} \le\delta$$

With this formulation the spectrum is only compared with the components of the P-/N-type data that were sampled. We solve (12) using an iterative thresholding (IT) implementation (Bostock et al. 2012). The modified algorithm, CS_RQD_, is able to reconstruct data with RQD-sampled gradient-selected time domains as purely absorptive, frequency-discriminated, high resolution spectra.

Of course, NMR experiments that include pulse sequence elements that are generally known by the description of ‘sensitivity enhanced’ or ‘preservation of equivalent pathways (PEP)’ (Cavanagh et al. 1991) that result in the transfer of both orthogonal coherence components modulated by the chemical shift during an evolution period are also suited to RQD and can be reconstructed by CS_RQD_ in analogy to the approach described here for P-/N-type RQD data. This applies also to any single transition-to-single transition polarization transfer (ST)_2_PT experiments e.g. the [^1^H,^15^N]-TROSY implementations used in this work. Hence, any strict interpretation of the P-/N-type, gradient-selection or phase modulation terminologies employed throughout this contribution should be relaxed to encompass any of the latter experiment types.

## Methods

### NMR spectroscopy

NMR experiments were recorded on a Bruker Avance AVIII 800 spectrometer operating at a ^1^H frequency of 800 MHz, equipped with a 5 mm TXI HCN/z cryoprobe. Data were collected at 298 K on samples that varied in concentration from 0.4 mM for ^15^N-labeled RalA-GDP, 0.3 mM for *U*-[^2^H,^15^N] Ala-[^13^CH_3_] [^2^H,^13^C,^15^N] Ile δ1-[^13^CH_3_] Leu,Val-[*pro*-(R),(S)-^13^CH_3_,^12^CD_3_]-pSRII to 0.25 mM for *U*-[^2^H,^13^C,^15^N]-labeled S195A-human factor IX. Experiments were recorded as gradient-enhanced implementations of 2D [^1^H,^15^N]-BEST TROSY (Lescop et al. 2010), 3D [^1^H,^15^N]-BEST TROSY HNCACB (Solyom et al. 2013) and 4D HCCH NOESY (Tugarinov et al. 2005). The key acquisition parameters for each of the experiments that generated the spectra shown in the Figures are given in Tables S1–6. For comparative purposes the individual experiments within the 2D, 3D and 4D series were recorded for equal lengths of time.

### Time domain sampling

Evolution times in the indirect dimensions were either sampled in full or using NUS. The NUS sampling schemes were generated using ScheduleTool software (Maciejewski et al. n.d.) or custom written scripts and were either exponentially biased, based on estimates of the expected *R*_2_ values for the indirect dimensions ^1^H (4D), ^13^C (3D, 4D) and ^15^N (2D) or randomly sampled for the constant time ^15^N (3D) evolution period.

### Frequency discrimination

For data sets with fully sampled and full-component NUS sampled indirect time domains, frequency discrimination in each indirect dimension was obtained either in full quadrature for every sampled time point through recording of both components *i.e.* P-type and N-type components in the case of phase modulation and gradient coherence order selection (Davis et al. 1992) or cosine and sine modulated components for amplitude modulated dimensions in States-TPPI fashion (States et al. 1982; Marion and Wüthrich 1983). In the case of random quadrature detection (RQD), for every sampled time point, only one quadrature component for all indirect dimensions was recorded, reducing the size of the data matrix to 1/2 (2D), 1/4 (3D) or 1/8th (4D) of the hypercomplex matrix and enabling a corresponding increase in acquired time points compared to the same total size of the matrix using full-component NUS. The quadrature component that was recorded was selected in a random manner, using in-house written scripts. Control over the quadrature component to be recorded was obtained via the Bruker VCLIST utility in Topspin. Representative RQD sampling schemes for 2D and 3D experiments are shown in Fig. S1.

### Data processing

Fully sampled spectra were processed by Fourier transformation using the Azara software package (W. Boucher, unpublished) while the remaining RQD, NUS and RQD-NUS undersampled experiments were reconstructed using a modification of our in-house developed CS reconstruction methods (Bostock et al. 2012), using MATLAB and Python and based on the iterative thresholding procedure (IT) as described.

2D and 3D reconstructions were carried out on a multi-user server with 48 AMD 6174 cores with 192 GB RAM using the Python multiprocessing module to run reconstructions over multiple cores. 4D reconstructions were carried out on the Cambridge high performance computing Darwin cluster; each node consists of two 2.60 GHz, eight core, Intel Sandy Bridge E5-2670 processors (sixteen cores in total per node) with 64 GB of RAM (4 GB per core). Code was adapted to use the MPI for Python package (mpi4py) with the Open MPI library. Typical processing times are shown in Table S7.

### Display of spectra

Contour levels in the Figures were adjusted to enable a direct comparison of peak intensities between the different spectra in a figure taking into account variations in the number of scans.

## Results and discussion

### Amplitude-modulated data

**Fig. 1:**
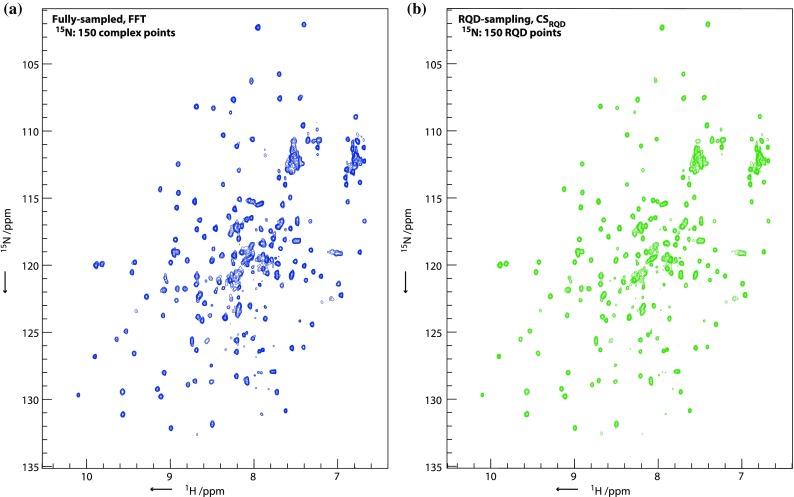
Reconstruction of a 2D SOFAST [^1^H,^15^N]-HMQC (Schanda and Brutscher 2005) for RalA·GDP showing the fully-sampled FFT reconstruction of a spectrum recorded with 150 complex points in the ^15^N dimension (**a**) in comparison to CS_RQD_ reconstruction of a spectrum recorded with 150 RQD points, i.e. the same $t_{{1,{ \hbox{max} }}}$ but selecting either the cosine or sine-modulated component at random for each time-increment, as suggested by Maciejewski et al. (2011) (**b**). For comparative purposes, **a** and **b** are recorded for the same total experiment time; **a** is recorded with ${\text{ns}} = 2$ and **b** with ${\text{ns}} = 4$. Due to the different number of scans, spectra are scaled to give the same maximum peak height in **a** and **b**. The two experiments are highly similar, with good reproduction of peak positions, shapes and intensities in the RQD spectrum (**b**). Acquisition parameters are given in Table S1

As proposed by Maciejewski et al. (2011) partial component sampling of quadrature components still allows the achievement of frequency discrimination, providing the quadrature components are sampled at random. Standard CS processing can reconstruct such spectra by constraining the reconstruction to the acquired cosine/sine components (${||\mathbf{Ax}} - {\mathbf{b}||}_{2}$ term in (6)). Similar to the previously suggested MaxEnt processing, CS reconstruction of such spectra efficiently suppresses artifacts from the RPD sampling and reproduces all the wanted peaks, generating a spectrum with frequency discrimination (Fig. 1).

### Phase-modulated data

**Fig. 2:**
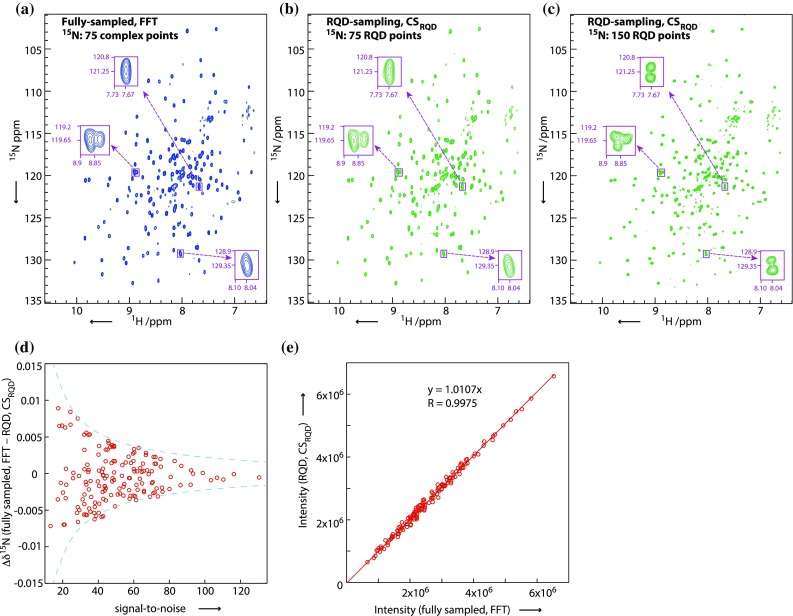
Comparison of a 2D gradient-enhanced [^1^H,^15^N]-TROSY of RalA·GDP showing the fully-sampled FFT reconstruction of an experiment recorded with 75 complex points in the ^15^N dimension (**a**) in comparison to the CS_RQD_ reconstruction of a dataset recorded with 75 RQD points, i.e. with the same maximum evolution time but selecting either the P- or N-type component for each time-increment at random (**b**). In **c** the time-saving from RQD is used to increase the resolution by recording 150 RQD points. For comparative purposes, **a**–**c** are recorded for the same total experiment time; for **b** this is achieved by doubling the number of scans (Table S2). The *purple boxes* show three enlarged regions which emphasize the increased resolution in **c** obtained through RQD sampling. **d**, **e** compare the ^15^N chemical shift positions and peak intensities from the FFT and CS_RQD_ spectra as *red circles*. The *blue lines* in **d** indicate the ^15^N chemical shift reproducibility of a fully sampled FFT reconstruction based on line width, acquisition time and signal-to-noise (Kontaxis et al. 2000). The *red circles* are all well within this reproducibility range. The higher resolution spectra were used for this analysis with 150 complex points (ns = 4) for the FFT reconstruction and 150 RQD points (ns = 8) for the CS_RQD_ reconstruction, with experiments recorded for equal amounts of time

As described in the theory section, reconstruction of partial component phase-modulated data requires modification of the standard CS algorithm to ensure the spectrum is constrained to the original P-/N-type data at the acquired data points (CS_RQD_). CS_RQD_ reconstruction of a 2D gradient-enhanced [^1^H,^15^N]-TROSY of the 165 residue G-protein RalA·GDP, acquired with RQD in the ^15^N dimension, is shown in Fig. 2. This is representative of the high spectral quality obtainable using CS_RQD_, demonstrating faithful reproduction of peak positions, intensities and line shapes when compared to a conventionally recorded FFT spectrum. Artifact levels in CS_RQD_ reconstructed RQD sampled data sets are generally very low and do not interfere with any spectral analysis. A substantial benefit of RQD sampling is the ability to increase the spectral resolution in the indirect dimension for a given experiment time (Fig. 2c). For an unbiased comparison all three spectra depicted in Fig. 2 were recorded for the same total amount of time. In the current comparison, RQD sampling enables doubling of the resolution (Fig. 2a–c, inserts). CS_RQD_ reconstruction of RQD sampled data faithfully reproduces peak positions and signal intensities (Fig. 2d, e).

**Fig. 3:**
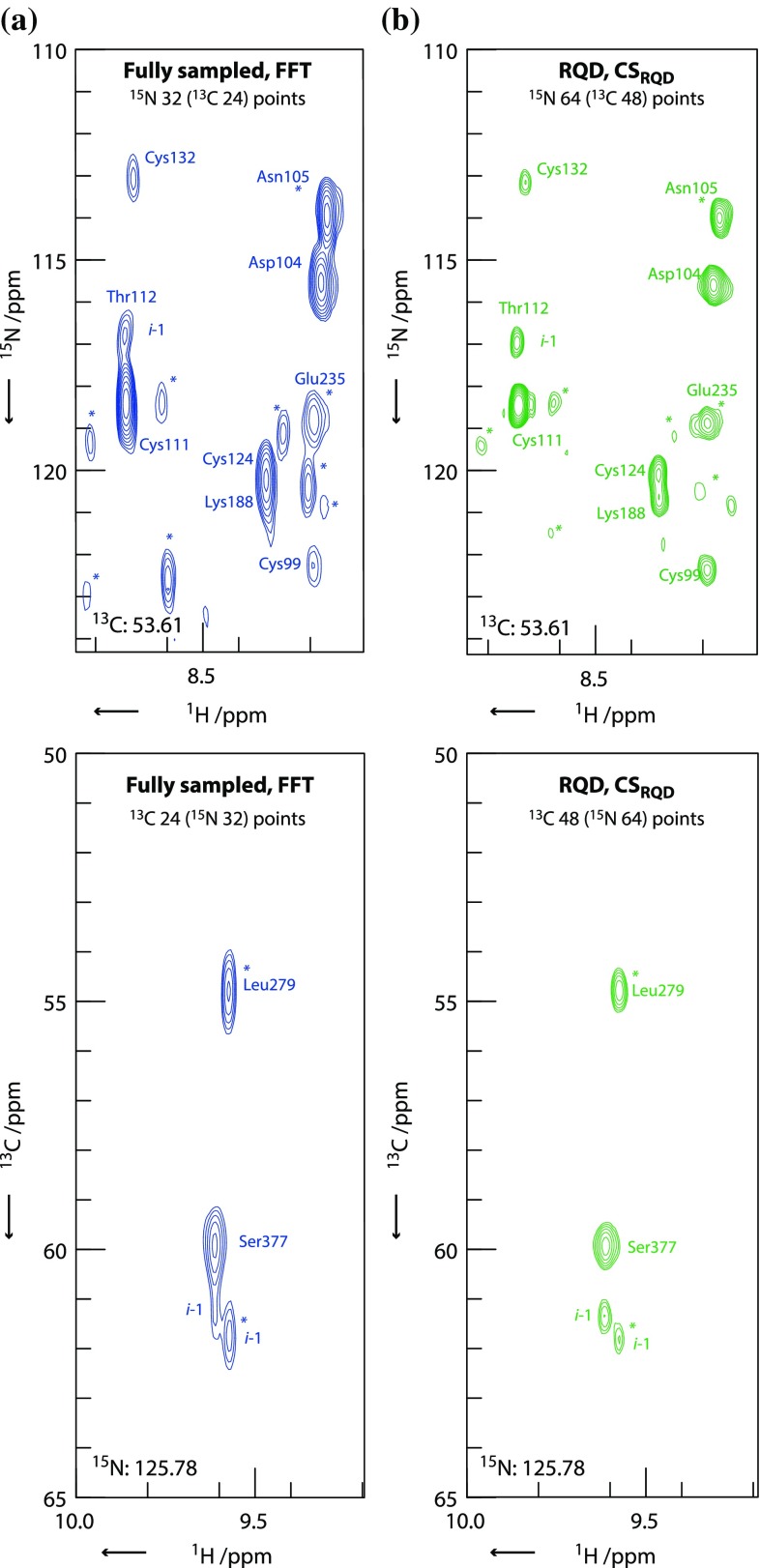
Selected 2D planes from the reconstruction of a 3D [^1^H,^15^N]-TROSY HNCA experiment recorded on S195A-human factor IX. **a** 2D [^1^H,^15^N] and [^1^H,^13^C] planes from a fully sampled, FFT reconstructed experiment with 32 × 24 complex points in the ^15^N and ^13^C dimensions respectively. **b** The equivalent planes from the CS_RQD_ reconstructed experiment where RQD sampling provides a factor of two saving in each indirect dimension, used here to increase the resolution to 64 × 48 time-points. Both experiments are recorded for the same total experiment time. Peaks indicated by an *asterisk* are breakthrough contributions from adjacent planes. The magnitude of the breakthrough peaks in the RQD spectrum is significantly reduced, consistent with the higher resolution of the RQD spectrum. Acquisition parameters are given in Table S3

Higher dimensional experiments, e.g. 3D, typically combine gradient-selection in one indirect dimension with amplitude modulation in another. RQD can be applied to both indirect dimensions as demonstrated for a 3D [^1^H,^15^N]-TROSY HNCA (Salzmann et al. 1998) recorded on S195A-human factor IX, a 297 amino acid, 33 kDa protein (Fig. 3) (Johnson et al. 2010). The time saving from RQD allows the resolution to be increased in both indirect dimensions in comparison with the fully sampled FFT experiment.

### Partial-component NUS

Although pure RQD may be of use for some higher dimensional ($n \ge 3$) experiments, such experiments are typically already recorded with full-component NUS to reduce data acquisition time and allow improvements in sensitivity and/or resolution. A key question is therefore whether RQD partial-component sampling combined with NUS (RQD-NUS) can outperform standard full-component NUS at a given resolution. This question has been considered theoretically with suggested benefits for partial-component NUS relative to pure NUS due to the increased randomization arising from randomization of the quadrature component in addition to the sampled time points (Schuyler et al. 2015). However, to our knowledge, no comparison in the context of real experimental data has been demonstrated and furthermore, considerations of the partial-component schedules (Schuyler et al. 2015) assume that both components generate an absorptive lineshape, equivalent to applying this approach to RQD-acquired amplitude modulated data (Maciejewski et al. 2011). In reality, this does not account for the challenge of handling the phase-twist lineshape introduced in RQD-acquired phase-modulated data.

**Fig. 4:**
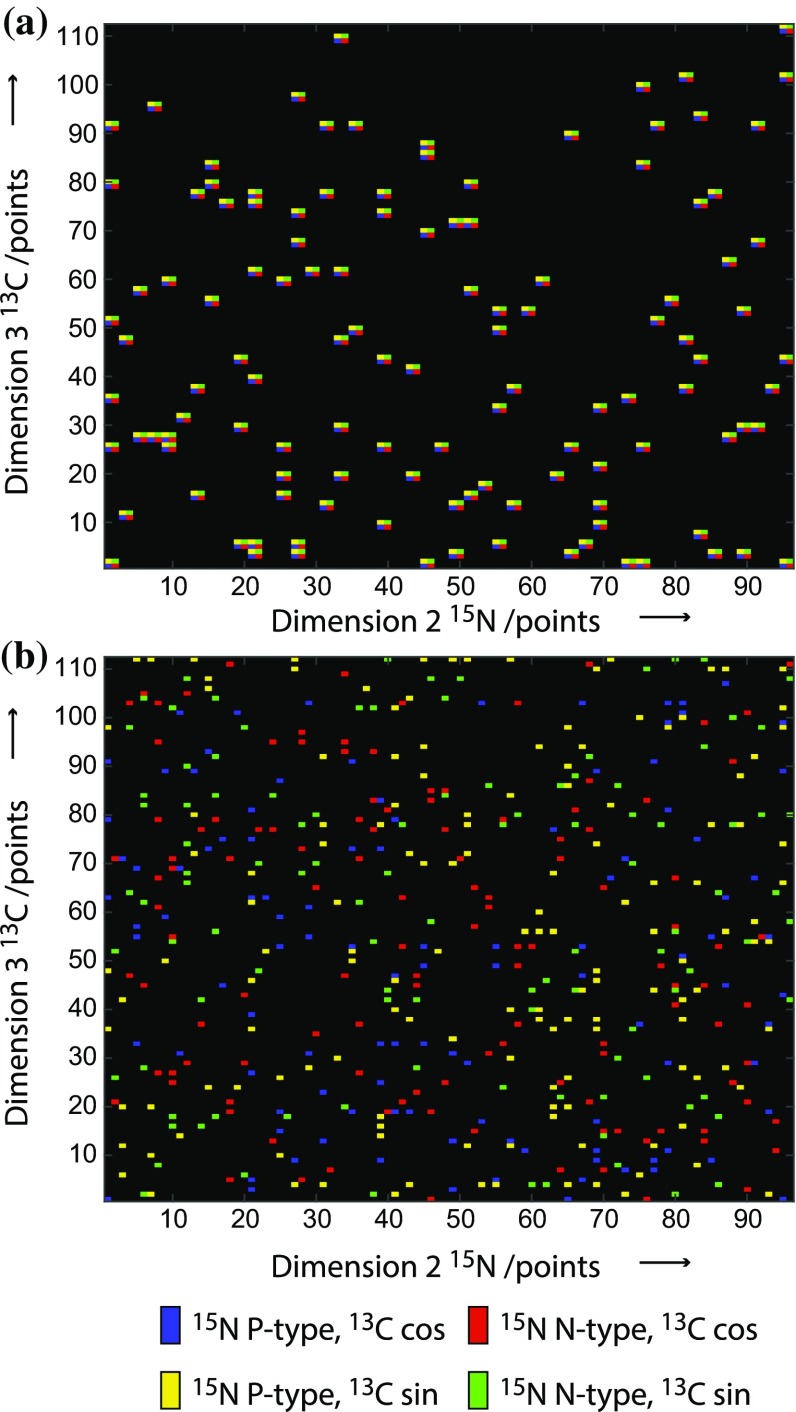
Comparison of NUS and RQD-NUS sampling schedules for 3D HNCACB data (Fig. 5b). Both schemes acquire the same total number of data points, however the RQD-NUS scheme is biased towards recording more time increments due to the factor of two reduction in quadrature component sampling required in each indirect dimension. Both schemes are drawn from the same exponential sampling distribution function. In **a** and **b**, the different quadrature components are represented with *different colours*, as indicated by the key

**Fig. 5:**
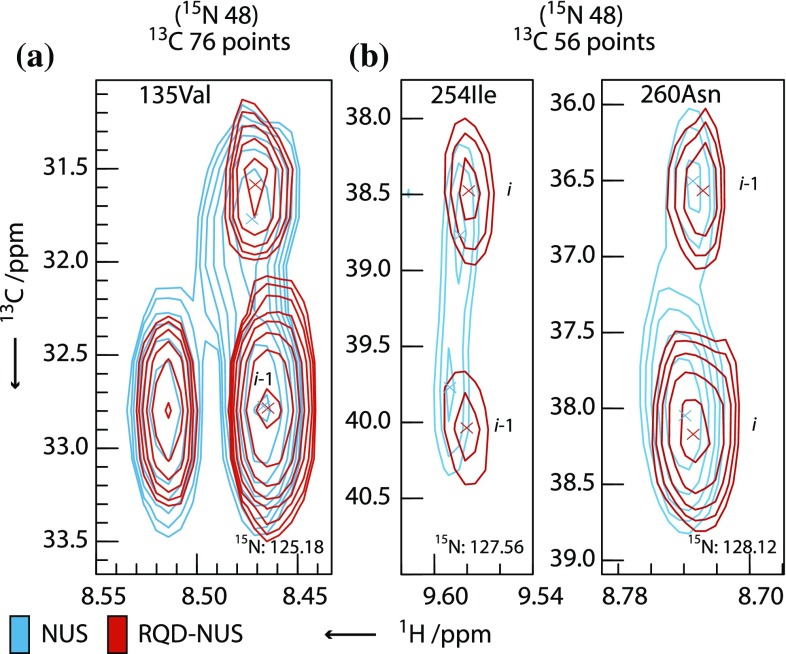
Selected 2D [^1^H,^13^C] planes from a 3D [^1^H,^15^N]-TROSY HNCACB experiment recorded on S195A-human factor IX using either full-component NUS with CS reconstruction or RQD-NUS sampling with CS_RQD_ reconstruction. **a** a 2D [^1^H,^13^C] plane from a CS-reconstructed experiment with 4.8 % sampling equivalent to a $t_{{1,{ \hbox{max} }}}$ of 48 × 76 complex points in the ^15^N and ^13^C dimensions, respectively, recorded with ns = 96. **b** Two 2D [^1^H,^13^C] planes from a CS-reconstructed experiment with 4.5 % sampling equivalent to a $t_{{1,{ \hbox{max} }}}$ of 48 × 56 complex points in the ^15^N and ^13^C dimensions, respectively recorded with ns = 192. Both NUS and RQD-NUS experiments are recorded for the same total experiment time. Acquisition parameters are given in Tables S4 and S5

Figure 4 shows an NUS sampling schedule for a 3D experiment compared with an RQD-NUS schedule of equivalent resolution. The schedules are displayed in total number of points with the different quadrature components shown in different colours. When considering sampling of time-points and quadrature components in an experiment, for illustrative purposes, these two aspects may be considered separately. In this view full-component NUS is biased towards full-quadrature sampling at the expense of time-point sampling. At the opposite extreme, RQD sampling is biased towards time-point sampling at the expense of sampling the quadrature components. For a full-component NUS schedule, the requirement to sample two components per time point in each indirect dimension reduces the coverage of time points for a given resolution; for an RQD-NUS schedule, two times as many time-points can be sampled per indirect dimension allowing a greater density of coverage. This provides greater flexibility in designing the schedule and may enable improved resolution due to the greater sampling density at longer time-points compared to an equivalent NUS schedule. Figure 5 compares peaks from a 3D [^1^H,^15^N] TROSY HNCACB experiment recorded either using NUS or RQD-NUS, sampled in both indirect dimensions to equivalent apparent $t_{{1,{ \hbox{max} }}}$ in each case (Tables S4 and S5). These examples demonstrate the increased resolving power of the RQD-NUS experiment, allowing peaks overlapped in the NUS spectrum to be distinguished for the same data acquisition time.

**Fig. 6:**
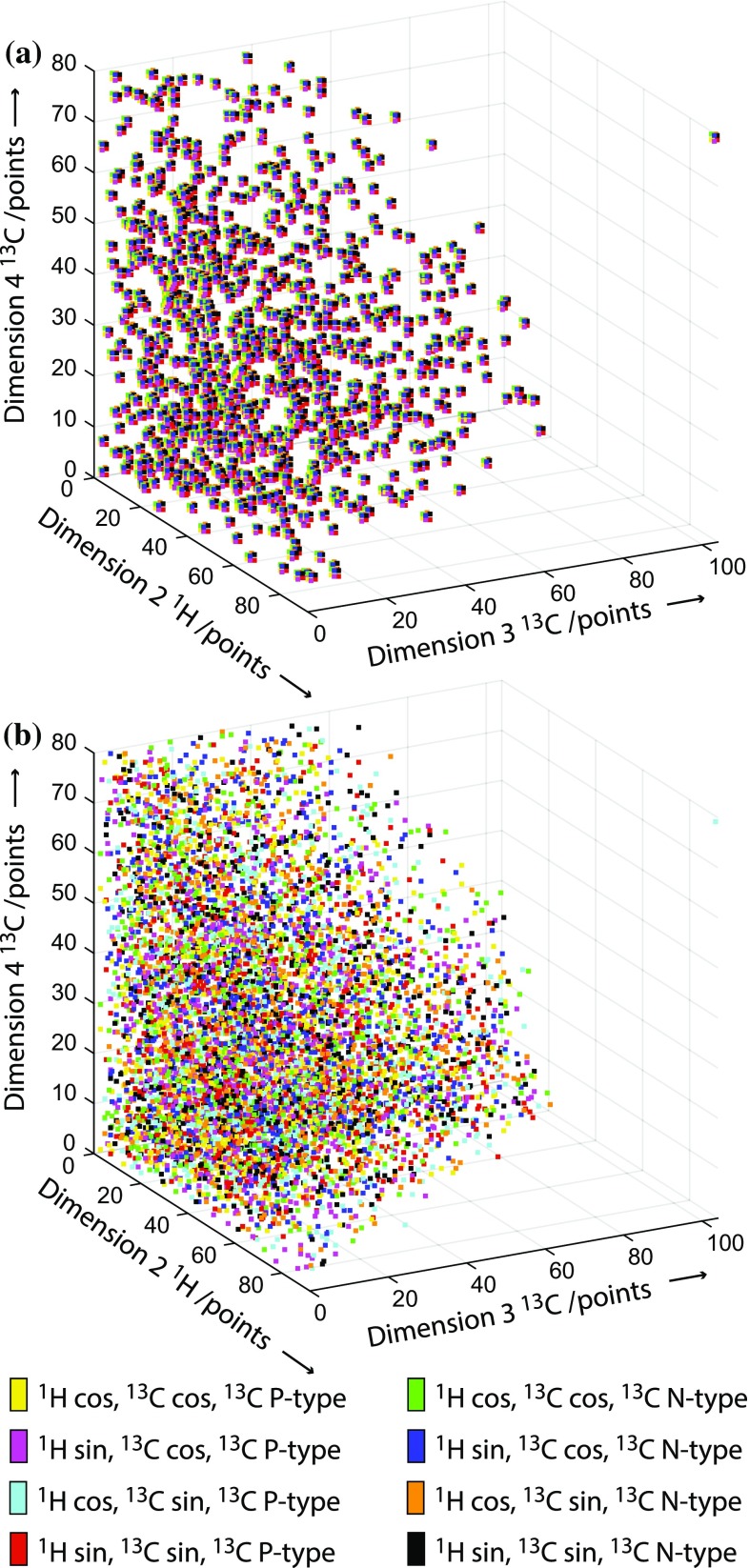
Comparison of NUS and RQD-NUS sampling schedules for a 4D HCCH (Fig. 7). **a** Full-component NUS and **b** RQD-NUS schedules with 8000 total points (1 % sampling). Both schedules are based on the same exponential sampling distribution function; the NUS schedule was generated by removing 87.5 % of the points from the RQD-NUS schedule and making the remaining 1/8th of the points into full-component quadrature points. The *axes* show total points in each dimension. The eight quadrature components are shown in different colours

**Fig. 7:**
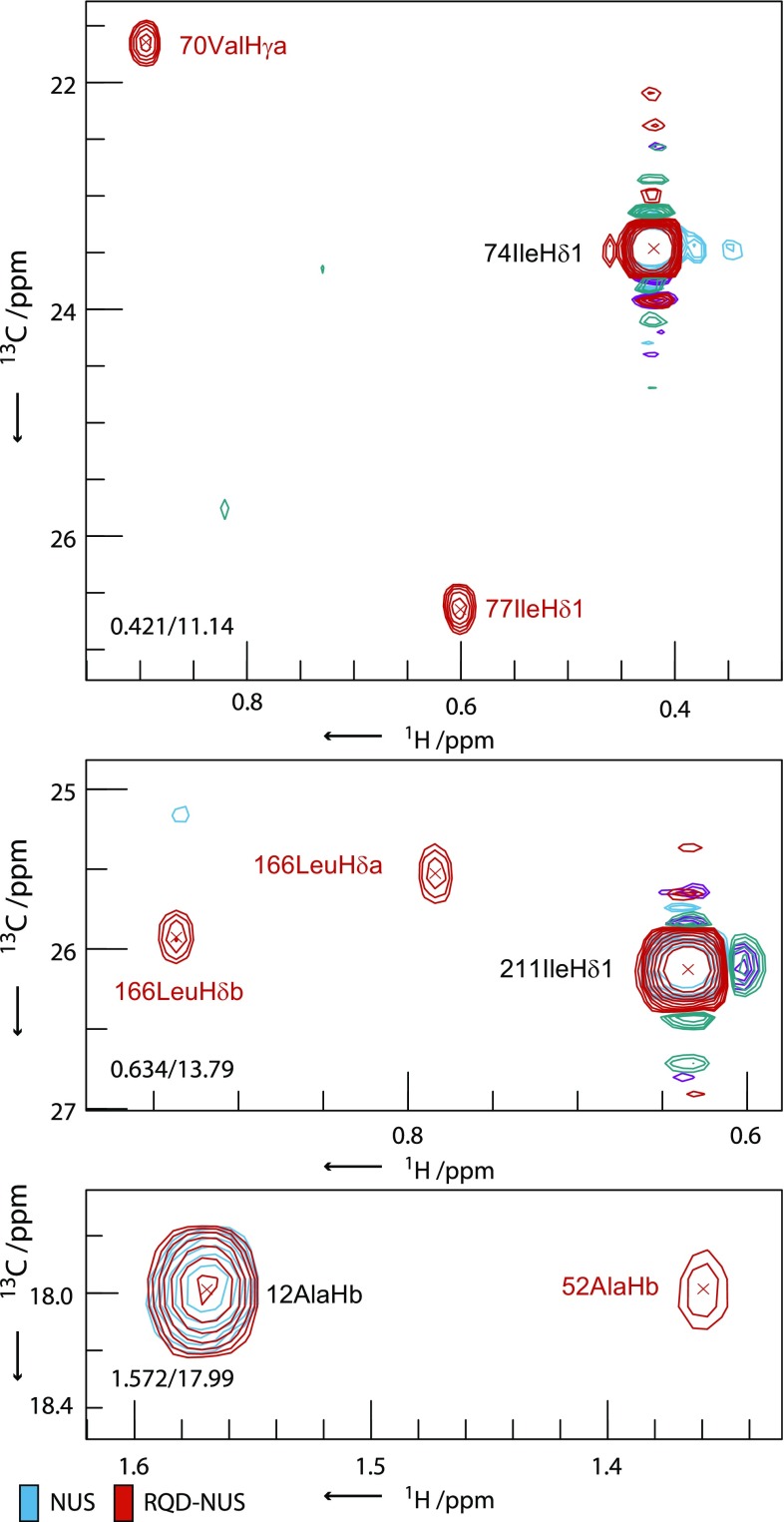
Selected 2D [^1^H,^13^C] planes ($$f_{1} ,f_{3} )$$ from the reconstruction of a gradient-enhanced 4D HCCH NOESY experiment recorded on ILVA methyl-protonated pSRII. The NUS-only experiment (*blue*/*purple*) is recorded with 1000 points from a matrix of 46 × 52 × 40 complex points (1 % sampling). The RQD-NUS (*red*/*green*) version was recorded for an equivalent time with 8000 time-points due to the factor of eight undersampling of quadrature components i.e. 1 % overall sampling. The cross peaks are indicated with red text. Full experiment details are given in Table S6. The sampling schedules used are illustrated in Fig. 6

For 3D spectra, RQD-NUS allows greater flexibility in point distribution when designing a sampling schedule, in this case resulting in improved resolution for a number of peaks; nevertheless in other parts of the spectrum where the resolution is not limiting there is no visible difference. However, as the dimensionality increases, the density of time-point coverage may need to be reduced substantially for full-component NUS in order to acquire a high resolution experiment in a given recording time. An example of a full-component NUS schedule for a 4D experiment is shown in Fig. 6a. The sampling fraction is 1 %, but since eight quadrature points must be acquired (two per indirect dimension) the reconstruction quality may be poor since so few time-points are characterised. In this situation reducing the bias towards quadrature components with partial-component NUS may provide greater benefits. RQD-NUS provides an eight-fold increase in time-point coverage as seen in Fig. 6b. This may be critical for successful spectral reconstruction at such low sampling density. Examples shown in Fig. 7 for a gradient-enhanced 4D HCCH-NOESY experiment with 1 % sampling compare the CS-NUS reconstruction with CS_RQD_ reconstruction of RQD-NUS data. The full-component NUS reconstruction fails to detect many of the important NOE cross peaks, which are essential for successful structure determination. The sampling distributions used for these reconstructions are shown in Fig. 6; for a fair comparison, the NUS schedule was generated by removing at random 87.5 % of the points from the RQD schedule and replacing these with full quadrature detection at each remaining time-point. The higher density of time-point sampling in the three indirect dimensions for the RQD schedule resulted in the higher performance of this method. Similar results were also observed using different distributions of NUS points (Fig. S2), indicating that this is not the effect of a single sampling distribution (Fig. S3).

## Conclusions

In conclusion, RQD partial-component sampling with CS reconstruction is a powerful method to remove the requirement for full quadrature detection in multidimensional NMR. RQD with CS_RQD_ is applicable to both phase and amplitude modulated data and its benefits are readily available to the full suite of modern NMR experiments. Such experiments are typically gradient-enhanced including the important TROSY-based sequences used for high molecular weight studies. RQD allows a 50 % reduction in the number of data points required per indirect dimension. This can significantly shorten higher dimensional experiments compared to their fully-sampled equivalents allowing the time saved to be converted into substantial resolution enhancements. When compared to full-component CS-NUS reconstructions recorded to equivalent apparent values for $t_{{1,{ \hbox{max} }}}$ the examples shown here for a 3D experiment demonstrate the potential of RQD to improve peak resolution. As the dimensionality increases, RQD-NUS schedules provide greater coverage of the time points in the $n - 1$ indirect dimensions, which may prove critical for successful spectral reconstruction. We expect further benefits for even higher dimensional experiments. Hence, RQD is of substantial benefit for biomolecular applications, particularly of large proteins or protein complexes, where signal overlap is a key limitation, and higher dimensional experiments are essential to NMR studies. RQD may also be used as a tool for time-saving in situations of high sensitivity e.g. for small molecules where the length of the experiment is determined by the required resolution (sampling limited). However, since RQD sampling diminishes the signal-to-noise ratio (SNR) by a factor of $\sqrt 2$ for every indirect dimension, shortening an experiment through RQD is only recommended in situations of good SNR. Of course RQD sampling is not limited to acquiring a single quadrature component at each time point; many other sampling scenarios can be envisaged where some time-points have full quadrature detection, others acquire one quadrature component and some time-points are skipped. Analysing the relative benefits of such schedules will be an important topic of future research. Although the experiments used to demonstrate RQD in this paper focus on proteins, the approach is general and will benefit any atomic resolution study that uses multidimensional NMR experiments.

## Electronic supplementary material

Below is the link to the electronic supplementary material.

Supplementary material 1 (DOCX 1382 kb)
