# Supplementary material for: Improving resolution in multidimensional NMR using random quadrature detection with compressed sensing reconstruction
Source: J Biomol NMR. 2016 Sep 20;68(2):67–77. doi: 10.1007/s10858-016-0062-9 (PMC5504140; doi:10.1007/s10858-016-0062-9)
Supplement: Supplementary file 1 — Supplementary material 1 (DOCX 1382 kb) [file 10858_2016_62_MOESM1_ESM.docx]

**Supplementary Information**

**Improving Resolution in Multidimensional NMR using Random Quadrature Detection with Compressed Sensing Reconstruction**

***Journal of Biomolecular NMR***

Mark J. Bostock,^[a]^ Daniel J. Holland,^[b]^ Daniel Nietlispach^[a],[*]^

^[a]^ Department of Biochemistry, University of Cambridge, 80 Tennis Court Road, Old Addenbrooke’s Site, Cambridge, CB2 1GA (UK)

^[b]^ Chemical and Process Engineering Department, University of Canterbury, Christchurch (New Zealand)

^[*]^ Correspondence should be addressed to Daniel Nietlispach

dn206@cam.ac.uk

**Supplementary Tables**

**Table S1.** Acquisition parameters for 2D SOFAST [^1^H,^15^N]-HMQC (Fig. 1)

|  | **Direct dimension** | **Fully sampled** | **RQD + fully sampled** |
| --- | --- | --- | --- |
|  | **(All Figures)** | **(Figure 1a)** | **(Figure 1b)** |
|  | **^1^H** | **^15^N** | **^15^N** |
| **Dimension** | Dim1 | Dim2 | Dim2 |
| **Sampling** | Uniform | Uniform | Uniform |
| **Number of time increments** | 512* | 150* | 150 |
| **t_1max_ (ms)** | 51 | 54.2 | 54.2 |
| **Frequency discrimination** | Quadrature | States-TPPI | RQD  (cos or sin) |
| **Number of scans** |  | 2 | 4 |
| **Spectral width (Hz)** | 10000 | 2778 | 2778 |
| **Offset (ppm)** | 4.75 | 118 | 118 |
| **Experiment time (min)** |  | 2.5 | 5 |
| **Processing/reconstruction** | FFT | FFT | CS_RQD_ |

**Table S2.** Acquisition parameters for 2D [^1^H,^15^N]-TROSY (Fig. 2)

|  | **Direct dimension** | **Fully sampled** | **RQD + fully sampled** | **RQD + fully sampled** |
| --- | --- | --- | --- | --- |
|  | **(All Figures)** | **(Figure 2a)** | **(Figure 2b)** | **(Figure 2c)** |
|  | **^1^H** | **^15^N** | **^15^N** | **^15^N** |
| **Dimension** | Dim1 | Dim2 | Dim2 | Dim2 |
| **Sampling** | Uniform | Uniform | Uniform | Uniform |
| **Number of time increments** | 512* | 75* | 75 | 150 |
| **t_1max_ (ms)** | 51 | 27.4 | 27.4 | 54.4 |
| **Frequency discrimination** | Quadrature | P/N | RQD (P or N) | RQD (P or N) |
| **Number of scans** |  | 4 | 8 | 4 |
| **Spectral width (Hz)** | 10000 | 2778 | 2778 | 2778 |
| **Offset (ppm)** | 4.75 | 118 | 118 | 118 |
| **Experiment time (min)** |  | 3 | 3 | 3 |
| **Processing/reconstruction** | FFT | FFT | CS_RQD_ | CS_RQD_ |

**Table S3.** Acquisition parameters for 3D [^1^H,^15^N]-TROSY HNCA (Fig. 3)

|  | **Direct dimension** |  | **Fully sampled** | |  | **RQD** | |
| --- | --- | --- | --- | --- | --- | --- | --- |
|  | **^1^H** |  | **^15^N** | **^13^C** |  | **^15^N** | **^13^C** |
| **Dimension** | Dim1 |  | Dim2 | Dim3 |  | Dim2 | Dim3 |
| **Time-point sampling** | Uniform |  | Uniform | |  | Uniform | |
| **Overall sampling** |  |  | 100% | |  | 25% | |
| **Number of time increments** | 512* |  | 768 | |  | 704 | |
| **Max. t_1_ point** |  |  | 32 | 24 |  | 64 | 48 |
| **t_1max_ (ms)** | 51.2 |  | 11.5 | 4.2 |  | 23.0 | 8.3 |
| **Frequency Discrimination** | Quadrature |  | P/N | States-TPPI |  | RQD  (P or N) | RQD  (cos or sin) |
| **Number of scans** |  |  | 24 | |  | 24 | |
| **Spectral width (Hz)** | 10,000 |  | 2778 | 5848 |  | 2778 | 5848 |
| **Offset (ppm)** | 4.7 |  | 118 | 46 |  | 118 | 46 |
| **Experiment time (h)** |  |  | 12 | |  | 11 | |
| **Processing/reconstruction** | FFT |  | CS | CS |  | CS_RQD_ | CS_RQD_ |

**Table S4.** Acquisition parameters for 3D [^1^H,^15^N]-TROSY HNCACB (Fig. 5a)

|  | **Direct dimension** |  | **NUS** | |  | **RQD-NUS** | |
| --- | --- | --- | --- | --- | --- | --- | --- |
|  | **^1^H** |  | **^15^N** | **^13^C** |  | **^15^N** | **^13^C** |
| **Dimension** | Dim1 |  | Dim2 | Dim3 |  | Dim2 | Dim3 |
| **Time-point sampling** | Uniform |  | NUS (4.8%) | |  | NUS (19.2%) | |
| **Overall sampling** |  |  | 4.8% | |  | 4.8% | |
| **Number of time increments** | 512* |  | 176 | |  | 704 | |
| **Max. t_1_ point** |  |  | 48 | 76 |  | 48 | 76 |
| **t_1max_ (ms)** | 51.2 |  | 17.2 | 5.7 |  | 17.2 | 5.7 |
| **Frequency Discrimination** | Quadrature |  | P/N | States-TPPI |  | RQD  (P or N) | RQD  (cos or sin) |
| **Number of scans** |  |  | 96 | |  | 96 | |
| **Spectral width (Hz)** | 10,000 |  | 2778 | 13333 |  | 2778 | 13333 |
| **Offset (ppm)** | 4.7 |  | 118 | 46 |  | 118 | 46 |
| **Experiment time (h)** |  |  | 11 | |  | 11 | |
| **Processing/reconstruction** | FFT |  | CS | CS |  | CS_RQD_ | CS_RQD_ |

**Table S5.** Acquisition parameters for 3D [^1^H,^15^N]-TROSY HNCACB (Fig. 5b)

|  | **Direct dimension** |  | **NUS** | |  | **RQD-NUS** | |
| --- | --- | --- | --- | --- | --- | --- | --- |
|  | **^1^H** |  | **^15^N** | **^13^C** |  | **^15^N** | **^13^C** |
| **Dimension** | Dim1 |  | Dim2 | Dim3 |  | Dim2 | Dim3 |
| **Time-point sampling** | Uniform |  | NUS (4.46%) | |  | NUS (17.8%) | |
| **Overall sampling** |  |  | 4.46% | |  | 4.46% | |
| **Number of time increments** | 512* |  | 120 | |  | 480 | |
| **Max. t_1_ point** |  |  | 48 | 56 |  | 48 | 56 |
| **t_1max_ (ms)** | 51.2 |  | 17.2 | 4.2 |  | 17.2 | 4.2 |
| **Frequency Discrimination** | Quadrature |  | P/N | States-TPPI |  | RQD  (P or N) | RQD  (cos or sin) |
| **Number of scans** |  |  | 192 | |  | 192 | |
| **Spectral width (Hz)** | 10,000 |  | 2778 | 13333 |  | 2778 | 13333 |
| **Offset (ppm)** | 4.7 |  | 118 | 46 |  | 118 | 46 |
| **Experiment time (h)** |  |  | 15 | |  | 15 | |
| **Processing/reconstruction** | FFT |  | CS | CS |  | CS_RQD_ | CS_RQD_ |

**Table S6.** Acquisition parameters for gradient-enhanced 4D HCCH NOESY (Fig. 7)

|  | **Direct dimension** |  | **NUS** | | |  | **RQD-NUS** | | |
| --- | --- | --- | --- | --- | --- | --- | --- | --- | --- |
|  | **^1^H** |  | **^1^H** | **^13^C** | **^13^C** |  | **^1^H** | **^13^C** | **^13^C** |
| **Dimension** | Dim1 |  | Dim2 | Dim3 | Dim4 |  | Dim2 | Dim3 | Dim4 |
| **Time-point sampling** | Uniform |  | NUS (1%) | | |  | NUS (8%) | | |
| **Overall sampling** |  |  | 1% | | |  | 1% | | |
| **Number of time increments** | 512* |  | 1000 | | |  | 8000 | | |
| **Max. t_1_ point** |  |  | 46 | 52 | 40 |  | 46 | 52 | 40 |
| **t_1max_ (ms)** | 51.2 |  | 28.8 | 21.0 | 16.1 |  | 28.8 | 21.0 | 16.1 |
| **Frequency discrimination** | Quadrature |  | States-TPPI | States-TPPI | P/N |  | RQD (cos or sin) | RQD (cos or sin) | RQD (P or N) |
| **Number of scans** |  |  | 40 | | |  | 40 | | |
| **Spectral width (Hz)** | 10000 |  | 1600 | 2480 | 2480 |  | 1600 | 2480 | 2480 |
| **Offset (ppm)** | 4.75 |  | 0.78 | 21.989 | 21.989 |  | 0.78 | 21.989 | 21.989 |
| **Experiment time (h)** |  |  | 110 | | |  | 110 | | |
| **Processing/reconstruction** | FFT |  | CS | CS | CS |  | CS_RQD_ | CS_RQD_ | CS_RQD_ |

**Table S7.** Representative processing times for NUS and RQD experiments

| **Dimensions** | **Experiment-type** | **Cores** | **Time** |
| --- | --- | --- | --- |
| **2D** | RQD | 4 | 105 s |
| **3D** | NUS | 12 | 1.5 h |
|  | RQD | 8 | 7 h |
|  | RQD-NUS | 12 | 10 h |
| **4D** | NUS | 64 | 28 h |
|  | RQD-NUS | 256 | 46 h |

**Supplementary Figures**


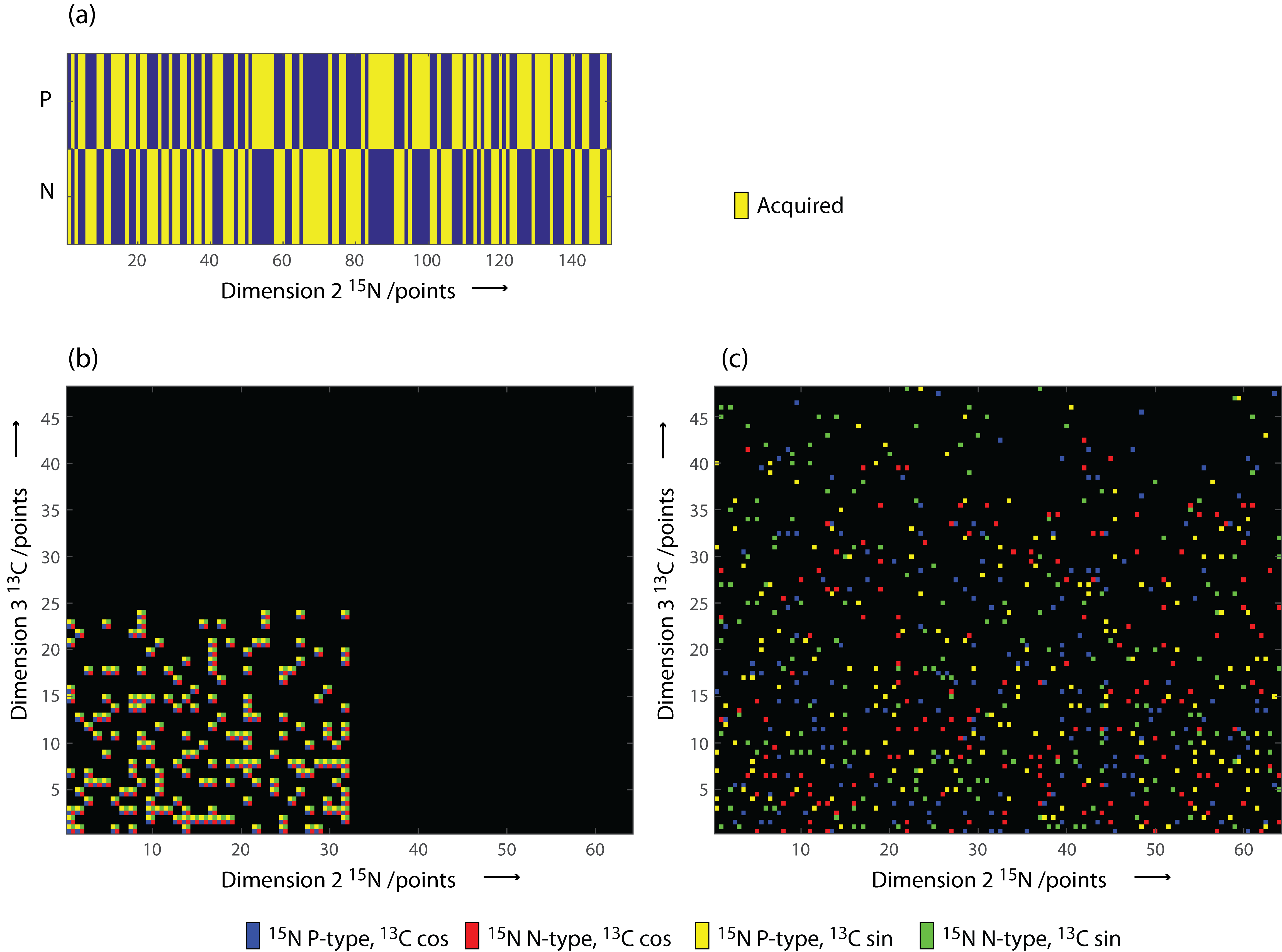


**Fig. S1**

Representative sampling schedules for 2D and 3D experiments. (a) shows an RQD schedule for a 2D experiment with acquired points indicated in yellow. For each time increment in the indirect ^15^N dimension (represented here as complex points), either the P or N point is acquired. The schedule was used for the spectrum shown in Figure 2c. (b) and (c) compare NUS and RQD-NUS schedules respectively for 3D experiments where the time-saving of RQD is used to extend the resolution in both indirect dimensions. For the NUS experiment each complex pair requires four quadrature components: P-type (^15^N), cos (^13^C); N-type (^15^N), cos (^13^C); P-type (^15^N), sin (^13^C); N-type (^15^N), sin (^13^C). These are represented respectively as blue, red, yellow and green squares. In contrast, in the RQD experiment, only one quadrature component is required for each complex pair, resulting in a factor of two undersampling in both the ^15^N and ^13^C indirect dimensions. For equal overall experiment times this allows a doubling of the resolution in both indirect dimensions allowing sampling out to 64 × 48 time-points in the RQD-NUS experiment, compared to 32 × 24 complex time-points in the NUS-only experiment.


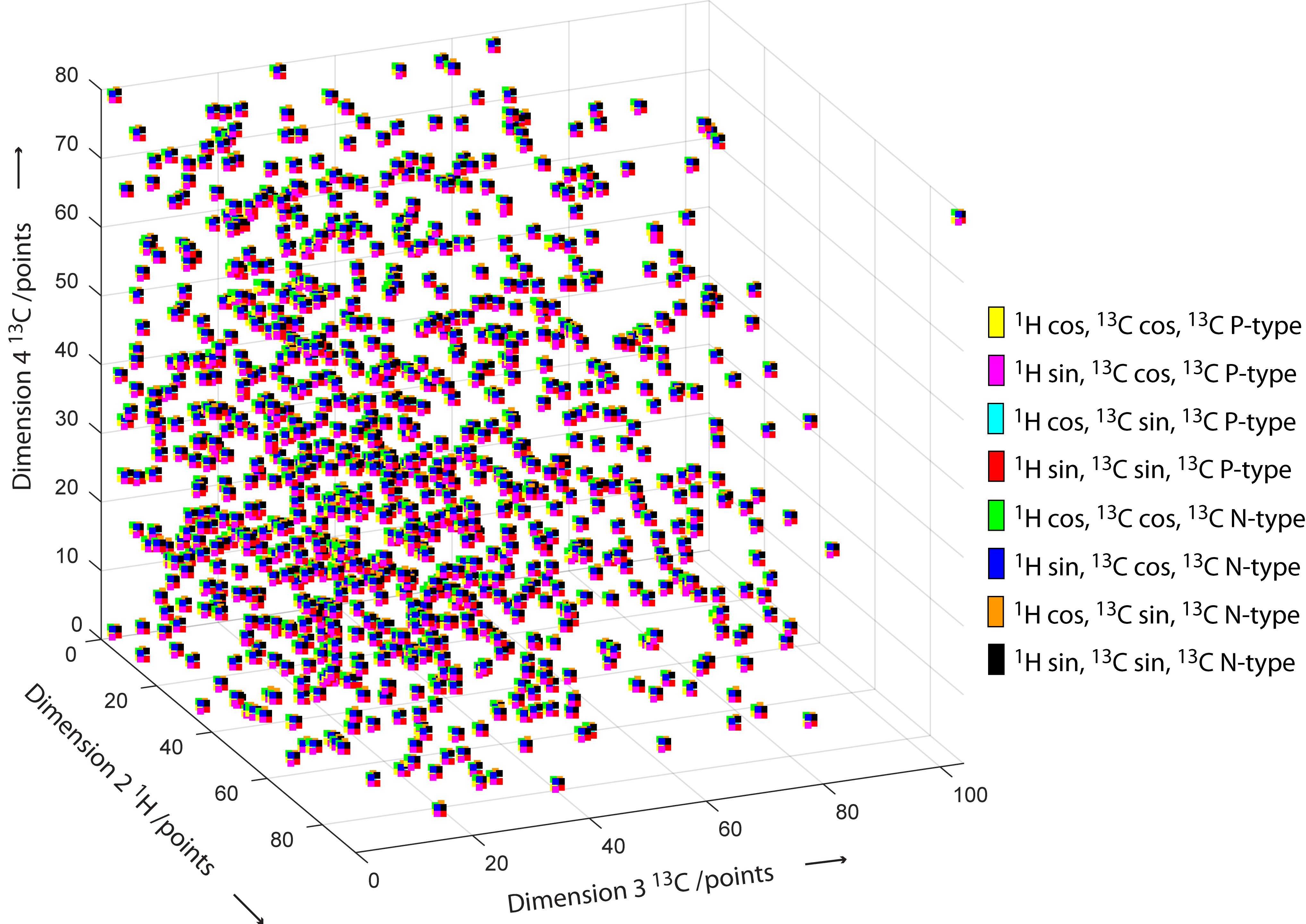


**Fig. S2**

An alternative NUS sampling scheme for a 4D HCCH experiment (Fig. S3). Similar to the NUS schedule in Fig. 6a, 8000 total points are recorded using full-component sampling, however a different exponential sampling function is used, resulting in the acquired points spreading out to higher time-increments in each of the three indirect dimensions. The eight quadrature components are shown in different colours.

**
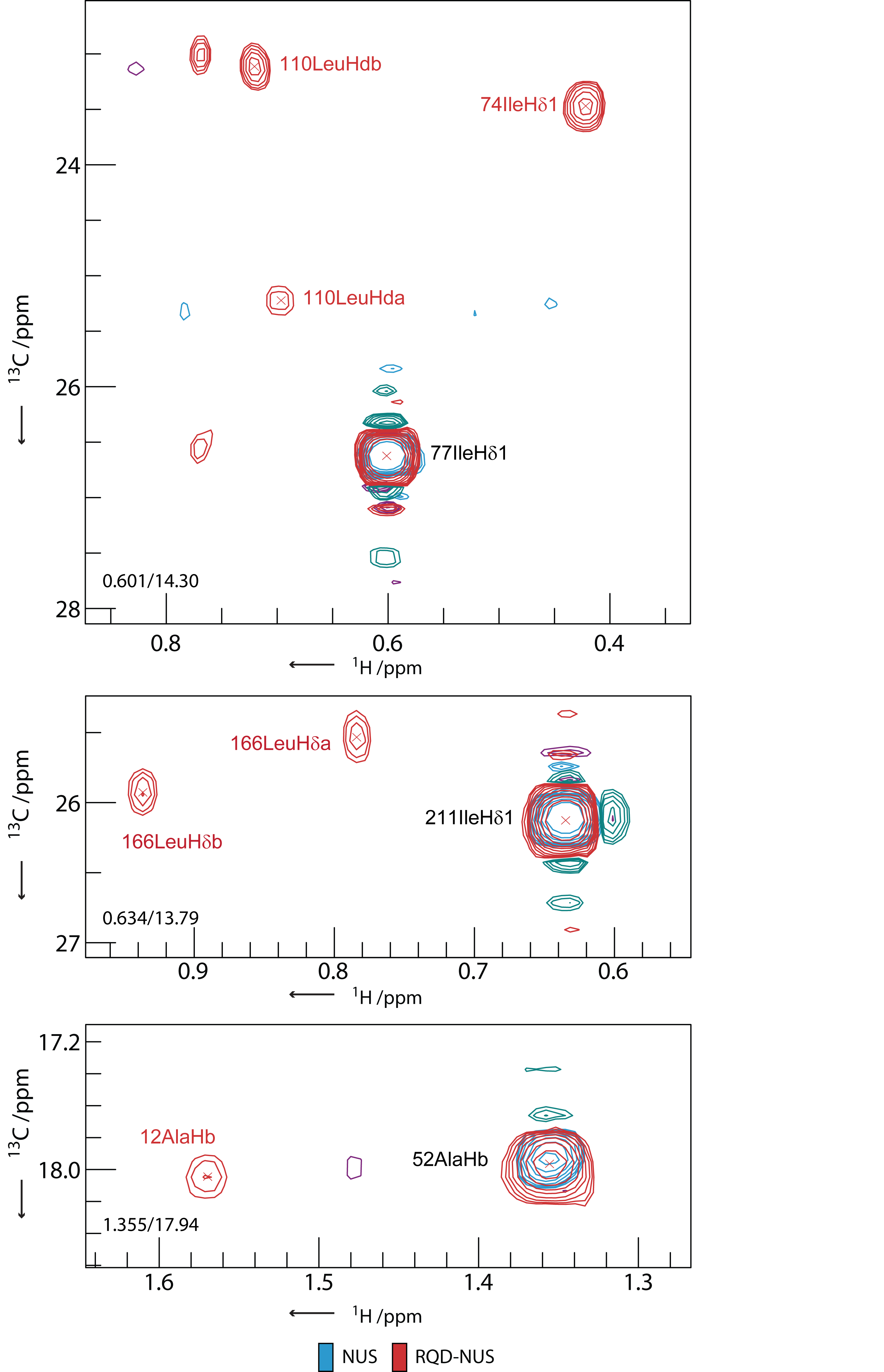
**

**Fig. S3**

Comparison of an NUS 4D HCCH experiment with an RQD-NUS experiment. Selected 2D [^1^H,^13^C] planes ($f_{1},f_{3})$ from the reconstruction of a gradient-enhanced 4D HCCH NOESY experiment recorded on ILVA methyl-protonated pSRII. The full-component NUS experiment uses the sampling schedule shown in Fig. S2. The RQD-NUS experiment uses the sampling schedule shown in Fig. 6b and is recorded using the identical pulse sequence as used in Fig. 7 (main text). The NUS-only experiment (blue/purple) is recorded with 1000 time-points in quadrature from a matrix of 46 × 52 × 40 complex points (1% sampling). The RQD-NUS (red/green) version was recorded for an equivalent time with 8000 time-points due to the factor of eight undersampling of quadrature components i.e. 1% overall sampling (Table S6). Cross peaks are indicated in red text.
